# Facile methodology of nanoemulsion preparation using oily polymer for the delivery of poorly soluble drugs

**DOI:** 10.1007/s13346-019-00703-5

**Published:** 2019-12-19

**Authors:** Johanna Wik, Kuldeep K. Bansal, Tatu Assmuth, Ari Rosling, Jessica M. Rosenholm

**Affiliations:** 1 Pharmaceutical Sciences Laboratory, Faculty of Science and Engineering, Åbo Akademi University, 20520 Turku, Finland; 2 Laboratory of Polymer Technology, Centre of Excellence in Functional Materials at Biological Interfaces, Åbo Akademi University, Biskopsgatan 8, 20500 Turku, Finland

**Keywords:** Nanoemulsion, Poly(δ-decalactone), Polymeric nanoemulsion, Stability, Aqueous solubility

## Abstract

Aqueous solubility of an active pharmaceutical ingredient (API) is a determining factor that has a direct impact on formulation strategies and overall bioavailability. Fabrication of nanoemulsions of poorly soluble drugs is one of the widely utilized approaches to overcome this problem. However, thermodynamic instability and tedious manufacturing processes of nanoemulsions limit their clinical translation. Therefore, this study was focused on circumventing the abovementioned hurdles by utilizing the polymer as an oil phase, instead of conventional oils. The nanoemulsion was prepared via a facile low-energy nanoprecipitation method using renewable poly(δ-decalactone) (PDL), as an oil phase and Pluronic F-68 as surfactant. The prepared nanoemulsions were characterized in terms of size, drug encapsulation efficiency, stability, and toxicity. Five different hydrophobic drugs were utilized to evaluate the drug delivery capability of the PDL nanoemulsion. The prepared nanoemulsions with sizes less than 200 nm were capable to enhance the aqueous solubility of the drugs by 3 to 10 times compared with the well-established Pluronic F-68 micelles. No phase separation or significant changes in size and drug content was observed with PDL nanoemulsions after high-speed centrifugation and 3 months of storage at two different temperatures (20 °C and 50 °C). PDL nanoemulsions were found to be non-heamolytic up to concentrations of 1 mg/mL, and the cell cytotoxicity studies on MDA-MB-231 and MEF cells suggest a concentration and time-dependent toxicity, where the PDL polymer itself induced no cytotoxicity. The results from this study clearly indicate that the PDL polymer has a tremendous potential to be utilized as an oil phase to prepare stable nanoemulsions via a facile methodology, ultimately favouring clinical translations.

**Electronic supplementary material:**

The online version of this article (10.1007/s13346-019-00703-5) contains supplementary material, which is available to authorized users.

**Graphical abstract:**
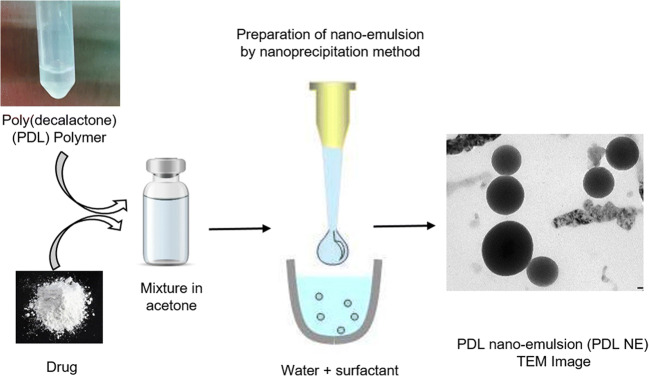
TOC graphic

## Introduction

Nanoemulsions are defined as dispersions of nanosized droplets of an immiscible liquid in another immiscible liquid, usually being oil and water, with the aid of surfactants [1]. Nanoemulsions are widely investigated in the field of pharmaceutics to enhance the aqueous solubility [2–4], bioavailability [5–7], and targeted delivery of drugs [8–11], as well as in food [12–14], cosmetics [15, 16], and agrochemical industries [17, 18]. Despite the several advantages it holds, the applicability of nanoemulsions is limited due to stability problems. Nanoemulsions, being thermodynamically unstable, could undergo Ostwald ripening along with flocculation and coalescence followed by creaming/sedimentation [19, 20]. Ostwald ripening is the major destabilization mechanism of nanoemulsions. It is a process where larger droplets grow on the expense of smaller droplets in the emulsion. Ostwald ripening is driven by the Kelvin effect, due to the difference in Laplace pressure, where small oil droplets have the property of increasing oil solubility to a larger extent than the larger droplets. The solubility of oil in the continuous phase is the main factor, which govern Ostwald ripening [7, 19–21]. Therefore, it has been suggested that the Oswald ripening process could be slowed down by the addition of a hydrophobic component into the oil, which could significantly decrease the coalescence rate and thus produce a stabilized nanoemulsion. Nanoemulsions prepared using high viscosity oils, such as long chain triglycerides (LCT), have shown not to experience Ostwald ripening and being physically stable for more than 3 months [7, 21].

In addition to the stability problem, expensive and tedious preparation methods of nanoemulsions are also a limiting factor in scale-up. Manufacturing of nanoemulsions usually involves two steps, where an initially prepared macroemulsion is converted into a nanoemulsion in a second step, using either high- or low-energy methods. However, mostly high-energy emulsification methods, which includes high-energy stirring, ultrasonic emulsification, high-pressure homogenization, and microfluidization are employed to get better control of droplet size and to avoid high temperature [7, 19, 22]. Moreover, preparation of a pseudoternary phase diagram is often needed to determine the optimum ratio of oil, surfactant, co-surfactant, and water [23, 24]. Last but not least, oxidation or rancidity of oil phase during storage is an another stability problem associated with nanoemulsions [25].

Recently, poly(decalactone) (PDL) polymer was reported as a viscous oily type of material, synthesised using a renewable monomer. The copolymer prepared using PDL was found to be biodegradable and less toxic in vitro and in vivo. Further, it was reported that the nanoemulsion of PDL could be readily obtained simply by low-energy nanoprecipitation method (spontaneous emulsification) thus avoiding use of sophisticated instruments to prepare nanoemulsions [26, 27]. As previously mentioned, there are several approaches to make nanoemulsions, but they are often difficult or expensive and require a phase diagram to obtain a stable emulsion. However, with polymeric nanoemulsions, a stable system with small droplet size could be produced without a phase diagram. It was anticipated that being an oily polymer with high hydrophobicity and viscosity, PDL could be able to make a stable nanoemulsion devoiding Ostwald ripening and rancidity, which is otherwise often observed with natural oils. Pluronic F-68 (also known as Poloxamer 188 or poly(ethylene oxide) (PEO) –b-poly- (propylene oxide) (PPO) –b- poly(ethylene oxide) (PEO) block copolymer) was chosen as a surfactant to stabilize the nanoemulsion due to its well-known stabilization property and FDA approval status for human use. This triblock copolymer has already been used in a few studies to make a stable nanoemulsion [28, 29].

Nanoemulsions are widely used for enhancing the aqueous solubility of drugs, and therefore the utility of this uniquely prepared nanoemulsion was evaluated by determining its ability to enhance the solubility of five different hydrophobic drugs: carvedilol, curcumin, cyclosporine A, griseofulvin, and prednisolone. The selected drugs differ in pharmacological action, intrinsic aqueous solubility, and molar mass, and thus could provide a better indication about the wide applicability of nanoemulsion as a drug carrier. Drug-loaded nanoemulsions were prepared via low-energy nanoprecipitation method and analysed for size, surface charge, drug content, stability, and cytotoxicity. Moreover, since Pluronic F-68 is capable of producing micelles, the results were compared with Pluronic micelles to assess the advantage of the nanoemulsions.

## Materials and methods

### Materials

δ-Decalactone (≥ 98%), 1,5,7-triazabicyclo[4.4.0]dec-5-ene (TBD) (98%), monomethoxy-PEG (Mn 5.0 kDa) (mPEG), propargyl alcohol (99%), cyclosporine A (≥ 98.5%), carvedilol (≥ 98%), curcumin (≥ 99.5%), prednisolone (≥ 99%), griseofulvin (97–102%), pluronic F68 (10% solution), and triton X-100 (BioXtra) were purchased from Sigma-Aldrich and used as received. All the solvents used were purchased from Fischer Scientific (UK), and MilliQ® water was used for preparing aqueous solutions.

### Methods

#### Synthesis of poly(δ-decalactone)

Poly(δ-decalactone) (PDL) polymer was synthesised according to the reported procedure via ring opening polymerisation (ROP) of monomer δ-decalactone in the absence of solvent [27]. Monomer (δ-decalactone, 10.00 g, 58.7 mmol) was added to a flask-containing initiator (propargyl alcohol, 0.03 g, 0.58 mmol), and stirred well to make a homogeneous mixture. Catalyst (TBD, 0.20 g, 1.4 mmol) was then added to the flask, and final mixture was then allowed to react for 8 h at room temperature. The obtained viscous liquid was later quenched by adding benzoic acid (0.35 g, 2.9 mmol) solution in acetone, precipitated in cold methanol (twice), and the residual solvent was evaporated under vacuum. Polymer propargyl-PDL was recovered as colourless viscous liquids with a yield of 8.03 g (80%).

^1^H NMR (500 MHz, CDCl_3_) δ (ppm) 4.86 (CH_2_–CH–O–C=O, m, 102H), 4.66 (C–CH_2_–O–CO, d, 2H), 3.69–3.45 (CH_2_–CH̲–OH̲, m, 4H), 2.46 (C ≡ CH, s, 1H), 2.44–2.12 (O–CO–CH_2_, m, 213H), 2.44–2.12 (CH̲_2_–CH̲_2_–CH– CH̲_2_, m, 616H), 1.35–1.05 (CH̲_2_–CH̲_2_–CH̲_2_–CH_3_, m, 631H), 0.86 (CH_2_–CH̲_3_, t, 305H).

^13^C NMR (126 MHz, CDCl_3_) δ (ppm) 173.50, 173.08 (CH–O–C̲O, CH_2_–O–C̲O), 74.82 (C̲H–C–CH_2_), 73.69, 71.34 (CH_2_–C̲H–O–CO, CH_2_–C̲H–OH), 73.55 (CH–C–CH_2_), 51.84 (CH–C–CH_2_), 37.48, 34.20 (CH–C̲H_2_–CH_2_), 36.84, 33.48 (C̲H_2_–CH–OH, C̲H_2_–CH–O–CO), 33.96, 34.45 (O–CO–C̲H_2_), 31.89, 31.66 (C̲H_2_–CH_2_–CH_3_), 25.35, 24.96 (CH–CH_2_–C̲H_2_), 22.65, 22.54 (O–CO–CH_2_– C̲H_2_), 20.80 (C̲H_2_–CH_3_), 14.01 (CH_2_–C̲H_3_).

Theoretical molecular weight (MW), 15.3 kDa, calculated MW by ^1^HNMR, 17.4 kDa.

MW by SEC: Mn, 9.4 kDa; Mw, 11.4 kDa; Mz, 14.4 kDa; PD, 1.21. Viscosity, 62.20 Pa.s.

#### Size exclusion chromatography

Size exclusion chromatography (Shimadzu, Germany) was used to determine the number-average molar mass (Mn), weight average molar mass (Mw), and mass distribution (polydispersity (PD), Mw/Mn) of the polymer. Tetrahydrofuran (THF) was used as mobile phase at 40 °C with a flow rate of 1 mL min − 1. The instrument was fitted with a low temperature evaporative light scattering detector (LT-ELSD) with AM GEL linear column and AM gel guard column (300 × 7.8 mm). Column calibration was done using narrow polystyrene standards of known Mn and PD in the range of 600–2300 kDa.

#### Nuclear magnetic resonance

The chemical structure of polymer was examined by proton nuclear magnetic resonance (^1^H-NMR) and carbon NMR (^13^CNMR) spectroscopy, using a Bruker NMR 500 MHz spectrometer (Bruker, Coventry, United Kingdom). Deuterated chloroform (CDCl_3_) was used as a solvent.

#### Viscosity

Viscosity of polymer was determined using TA instrument rheometer (AR 2000). Measurements were done at 23 °C, at shear rates ranging from 1 to 45 s^−1^. The viscosity was determined using rheology data advantage analysis software version 7.0 by fitting the data using viscosity vs rate.

#### Preparation methods of nanoemulsions

A nanoprecipitation method was used to prepare nanoemulsion using PDL polymer as oil and Pluronic F-68 as surfactant. Drug-loaded oil-in-water nanoemulsions were prepared by dissolving drug (5 mg) and polymer (PDL 25 mg) in solvent (acetone 1.5 ml). This organic mixture was then added dropwise to the milli Q water (3.5 ml), containing surfactant (Pluronic F-68, 1.5 ml) with stirring (1000 rpm). The solution was then stirred for at least 3 h at room temperature (open vial) to ensure the complete removal of organic solvent. The nanoemulsion was finally filtered through a membrane syringe filter (pore size, 0.45 μm) and used for further characterisation. Simultaneously, a similar solution was prepared for comparison purposes using Pluronic F-68 and drugs only. Blank nanoemulsion was prepared following similar process but without drug. Curcumin is light sensitive, and thus, the preparation of curcumin-loaded nanoemulsion was performed under dark.

#### Particle size and surface charge analysis

Globules size, polydispersity index, and surface charge of nanoemulsion were measured on a ZetaSizer NanoZS® (Malvern Instruments, UK). The light used by instrument is sourced from Helium -Neon laser with wavelength of 633 nm. Samples were diluted (50 μg/mL with respect to PDL) with MilliQ water and transferred into respective cuvettes for analysis. Measurements were performed at 25 °C, and data analysis was carried out using the Malvern ZetaSizer software version 7.12.

#### Drug content

All samples were analysed on Ultraviolet-visible (UV-Vis) NanoDrop 2000c spectrophotometer (Thermo Fisher Scientific, USA) except for cyclosporine samples. Prednisolone was analysed at λ_max_ 247 nm, carvedilol at λ_max_ 242 nm, griseofulvin at λ_max_ 295 nm, and curcumin at λ_max_ 423 nm. The concentrations of cyclosporine A was estimated using HPLC. The mobile phase used was water and acetonitrile (20:80%) with 0.05% of TFA in the acetonitrile. The temperature of column (Gemini-NX 3u C18 110A, 100 × 4.6 mm) was set to 50 °C. Flow rate was set to 0.7 ml/min, and absorbance was measured at 210 nm. The analysis was performed using a Merck Interface D-7000 Diode Array Detector, and samples were run for 7 min to determine the retention time of cyclosporine A. The drug concentrations was calculated using pre-prepared standard calibration curves and percentage drug content (DC%), and encapsulation efficiency (EE%) was established according to the reported formula [26].

#### Transmission electron microscopy (TEM)

TEM images were taken to confirm the size and to determine the surface morphology. Samples were imaged on TEM grids without staining. TEM images were taken for both empty and drug-loaded nanoemulsions, using a JEM 1400-Plus (JEOL Ltd., Tokyo, Japan). To perform the TEM observations, the nanoemulsion formulation was diluted with water (50 μg/mL with respect to PDL) and filtered through a membrane syringe filter (0.2 μm). A drop of the diluted nanoemulsion was then directly deposited on the copper grid and observed in TEM after drying.

#### Nanoemulsion stability study

The stability of nanoemulsion in terms of phase separation, size change, and drug degradation was evaluated by high-speed centrifugation (for force separation) and by storage at different temperature. Empty and prednisolone-loaded nanoemulsion was centrifuged for 30 min at 10,000 rpm, and phase separation was analysed visually.

For long-term stability studies, nanoemulsion loaded with prednisolone and cyclosporine A was selected. Nanoemulsion samples were stored at room temperature (20 ± 2 °C) and incubated at 50 ± 2 °C separately for 3 months. Samples were analysed visually for separation and for change in size, and drug content after every 30 days.

#### Ex vivo haemolytic study

The haemolytic study was performed according to reported methodology with minor modifications [26]. To prevent coagulation, 5 ml of human blood from an anonymous donor was drawn directly into a Na_2_-EDTA-coated tube. The blood was then centrifuged at 500 g for 5 min to separate the plasma and red blood cells (RBCs), and the plasma was discarded. RBCs were washed twice with 150 mM NaCl solution followed by one wash with phosphate buffer saline (PBS, pH 7.4). RBCs were diluted up to 5 times with PBS (pH 7.4) to make a stock suspension.

Nanoemulsion (50 mg/ml with respect to PDL containing 40 mg/mL of Pluronic F-68) prepared in PBS were further diluted to make 25, 12.5, and 1.25 mg/ml concentration in PBS. For each assay, 800 μl of nanoemulsions of all concentrations were added to 200 μl RBCs (from stock) to make 1 ml. Therefore, the stocks of 50, 25, 12.5, and 1.25 mg/ml, resulted in concentrations of 40, 20, 10, and 1 mg/ml of nanoemulsion. Positive control tubes were prepared by adding 800 μl of 1.25% solution of triton X-100 in 200 μl RBCs. Negative control tubes were prepared by adding 800 μl of PBS in 200 μl RBCs. The tubes were incubated at 37 °C for 1 h and 24 h. After incubation the tubes were carefully handled and centrifuged at 500 g for 5 min. From each tube, the supernatant was then analysed on UV-Vis spectroscopy to measure the absorbance of released haemoglobin at λ_max_ 542 nm. From the results of the UV-Vis spectroscopy, the percentage haemolysis was calculated.

#### Cell study

Cell growth media: MDA-MB-231 (human breast adenocarcinoma cells) and non-cancerous mouse embryonic fibroblasts (MEF) cells were cultured in Dulbecco’s Modified Eagle’s Medium (DMEM) supplemented with 10% fetal bovine serum, 2 mM l-glutamine, and 1% penicillin*-*streptomycin (v/v).

The cytotoxicity of nanoemulsion was evaluated using WST-1 cell viability assay (Roche Diagnostics, Mannheim, Germany) on MDA-MB-231 cells using reported procedure with minor modifications [30]. Briefly, 100 μl of cell stock suspension having concentration of 50,000 cells/ml was seeded into a 96-well plate and incubated for 24 h. The different concentrations of nanoemulsion, i.e. 0.25, 0.5, and 1 mg/ml from stock (5 mg/ml PDL in PBS containing 30 mg/ml Pluronic F-68 as stabilizer), were prepared in pre-warmed (37 °C) growth media. Similarly, only Pluronic F-68 was diluted in media to achieve the equivalent concentration used to prepare nanoemulsion. The cell media in 96-well plate was replaced after 24 h with nanoemulsion and Pluronic F-68 solutions. After 48 h and 72 h incubation at 37 °C, 5% CO_2_, 10 μl of WST-1 cell proliferation reagent was added, and the plate was incubated for additionally 2 h. The absorbance of samples was then read according to the manufacturer protocol (420–480 nm). The percentage cell proliferation was reported relative to untreated cells (100% viability). Similar procedure was followed to determine the toxicity of curcumin and curcumin-loaded nanoemulsion. Curcumin stock solution was prepared in DMSO to achieve the similar concentration of curcumin present in the nanoemulsion sample. Two different concentration of curcumin (i.e. 20 and 40 μg/mL) in cell media was prepared and incubated with cells for 48 h and 72 h to assess the cytotoxicity.

#### Statistical analysis

Statistical analysis was conducted by one-way ANOVA with Tukey’s multiple comparisons test using *P* < 0.05 (95% confidence interval) as a statistical significance threshold unless mentioned specifically. All statistical analysis was performed using graph-pad prism software (version-6) using *n* = 3. Statistical significance has been presented as extremely significant (****, *P* < 0.0001), extremely significant (***, *P* = 0.0001 to 0.001), very significant (**, *P* = 0.001 to 0.01), significant (*, *P* = 0.01 to 0.05), and not significant (ns, *P* ≥ 0.05).

## Results and discussion

### Synthesis and characterization of polymer

**Scheme 1:**
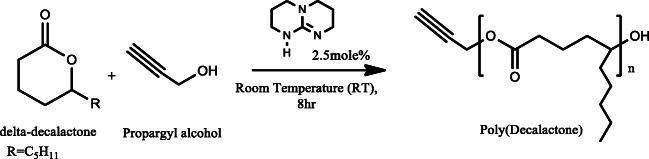
Ring opening polymerization of δ-decalactone to generate poly(decalactone) polymer using TBD as catalyst

**Fig. 1:**
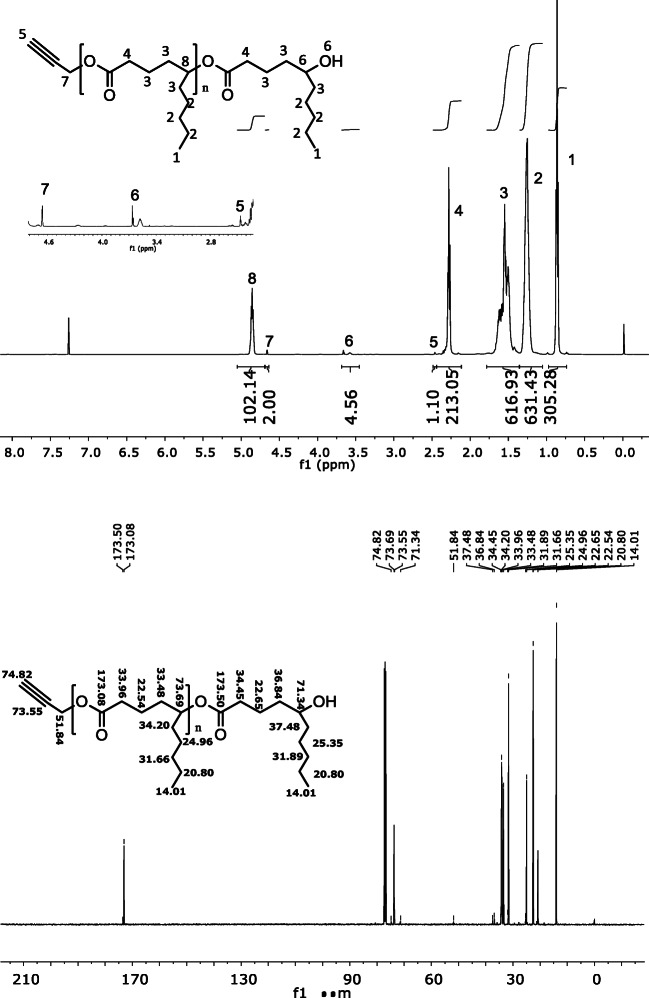
^1^HNMR and ^13^CNMR (bottom) of poly(decalactone) in CDCl_3_

**Fig. 2:**
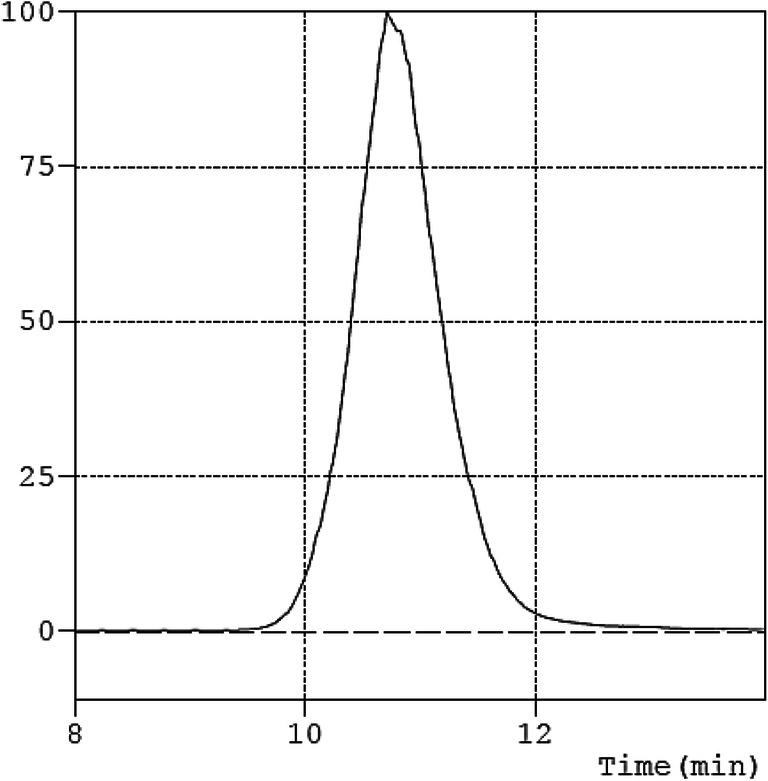
SEC trace of poly(decalactone) polymer using THF as eluent (with reference to polystyrene polymer as standard)

**Fig. 3:**
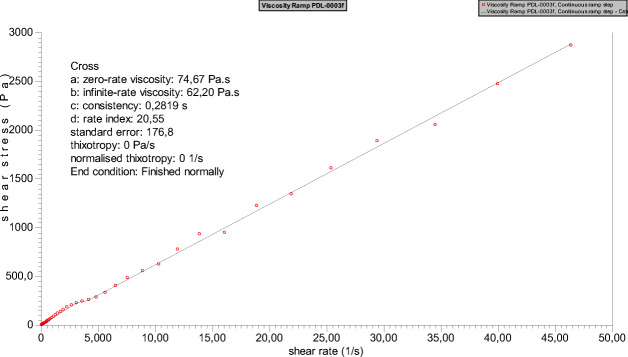
Shear rate versus shear stress curve of PDL polymer

The polymer was synthesized via well-known ROP route in bulk following a reported procedure using 100 as degree of polymerization (DP) (Scheme 1). δ-Decalactone is an FDA-approved flavouring agent and a candidate monomer for biomedical polymer applications. The synthesis methodology is straightforward, without a need of any special reaction setup and could be considered as an industrially friendly synthesis approach. The percentage conversion of monomer to polymer was calculated before purification via ^1^HNMR by integrating the peak at 4.2 (monomer peak) and 4.8 ppm (polymer peak), and was found to be 90%. The unconverted monomer and catalyst were then washed out by cold methanol to obtain pure polymer. Proton and carbon NMR of the polymer confirmed the synthesis and recovery of pure polymer, and the observed peaks matched with the reported values [27] (Fig. 1). The higher value of calculated MW of polymer (by ^1^HNMR) to theoretical MW can be attributed to the presence of homopolymer chain (initiated by alcohol other than propargyl alcohol) in the sample. The similar phenomena were reported earlier by Bansal et.al., where the presence of ring-opened monomer δ-decalactone was responsible for producing undesired homopolymer. Since the MW by NMR was calculated by number of protons at 4.8 ppm with respect to the peak of initiator at 4.6 ppm, such misinterpretation of MW is highly possible. SEC result suggested unimodal molecular weight distribution of polymer with low polydispersity index (Fig. 2) [27]. To establish an easier characterization parameter, we have also determined the viscosity of polymer to control batch-to-batch variability in polymer synthesis. It is well-known that polymer viscosity is directly proportional to its molecular weight, and therefore viscosity offers an easiest way to estimate consistency in polymer batches. The shear rate vs shear stress curve suggested that the PDL polymer is a Newtonian fluid, and the infinite rate viscosity was found to be 62.20 Pa.s (Fig. 3). Nevertheless, it was assumed that the presence of undesired homopolymer is not going to affect the nanoemulsion preparation, but will certainly avoid additional purification steps. In future studies, PDL polymer can be obtained without using initiator, and viscosity could be used as a characterization parameter.

### Preparation and characterization of nanoemulsion

In this study, the nanoemulsion was prepared by low-energy emulsification method (nanoprecipitation method), which is also known as spontaneous emulsification. Spontaneous migration of water miscible organic solvent (acetone) containing PDL (with or without drug) to the aqueous phase took place upon dropwise addition. This migration leaves the tiny droplets of PDL behind, which was immediately stabilized by Pluronic F-68 to produce a nanoemulsion.

**Fig. 4:**
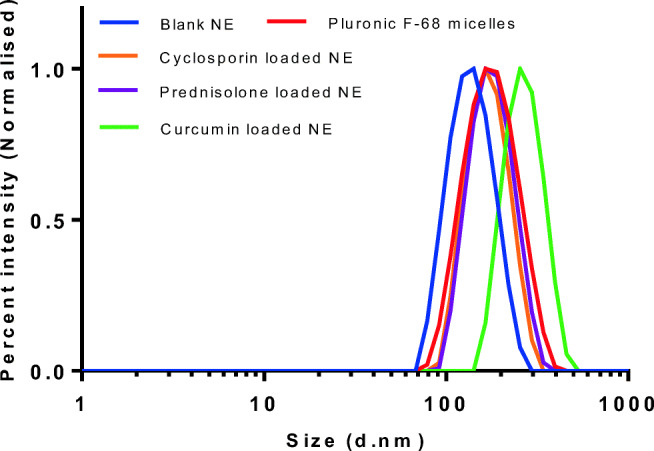
Size distribution by intensity for blank, drug-loaded nanoemulsion (NE) and Pluronic micelles

**Fig. 5:**
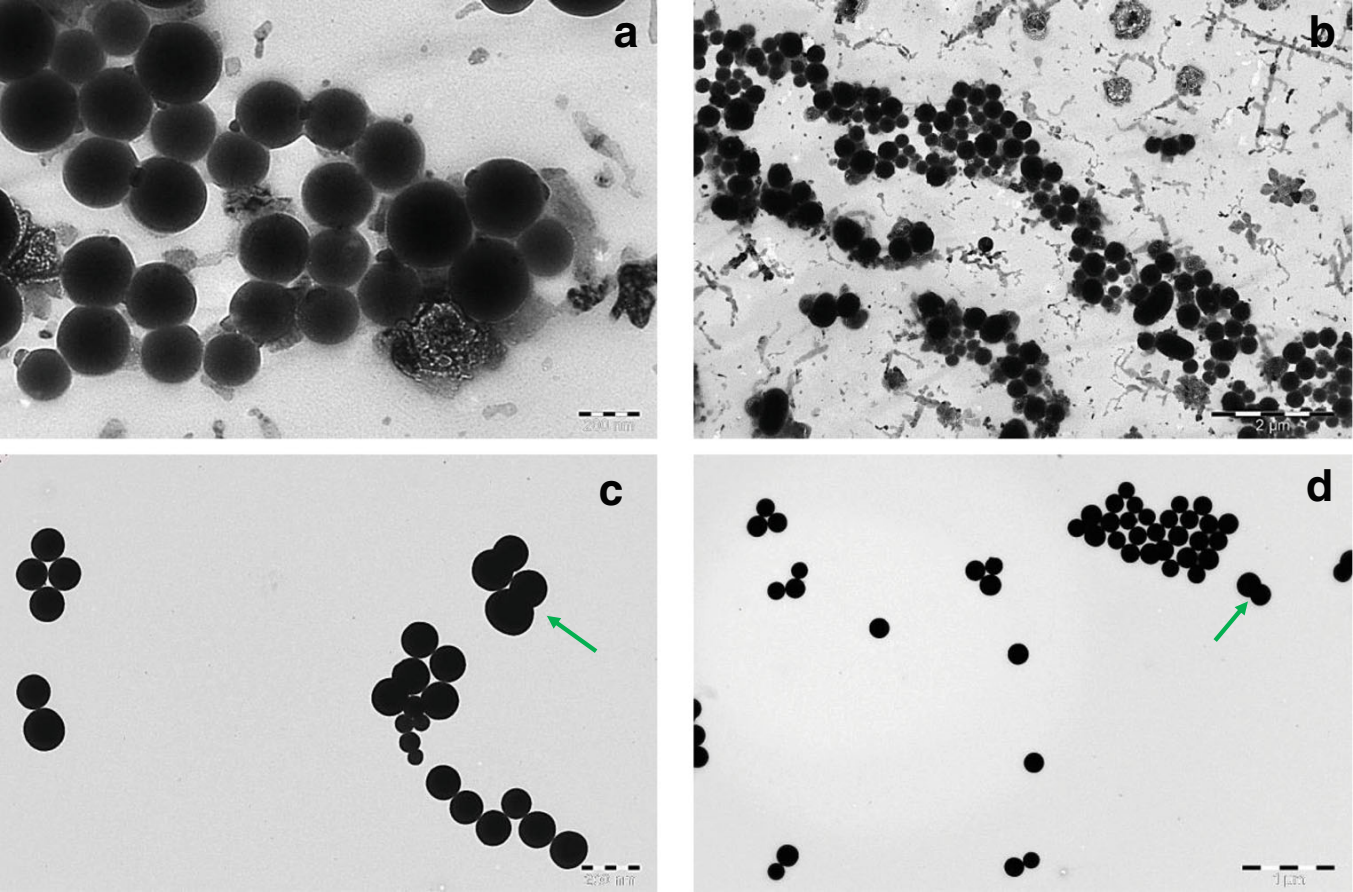
TEM images of blank nanoemulsion with scale bar **a** 200 nm, **b** 2 μm and curcumin-loaded nanoemulsion with scale bar **c** 200 nm and **d** 1 μm. Arrow represents fusion of globules

The droplet size in the emulsion was analysed by dynamic light scattering (DLS) and TEM after appropriate dilutions. The Z-average sizes obtained by DLS were less than 200 nm with low polydispersity index (PdI) except for curcumin-loaded samples, where the size observed was above 200 nm (Table 1, Fig. 4). It can be presumed from the results that the size and polydispersity of Pluronic micelles was reduced when the core was filled with PDL polymer. Reduction in Pluronic micelles size after addition of highly hydrophobic oily polymer suggested a strong interaction between PDL and PPO blocks, leading to the reduction in overall micelle size and PdI. A similar phenomenon has been observed earlier when hydrophobic compounds were loaded in Pluronic micelles [31, 32]. However, as expected, an increment in size was observed after loading of hydrophobic drugs within the nanoemulsion. The size and surface morphology of blank and curcumin-loaded nanoemulsion were further examined by TEM, and the images suggested that the emulsion droplets are spherical in shape with a size less than 200 nm (Fig. 5). However, a few fused globules are clearly visible in the TEM images of curcumin-loaded nanoemulsion, which could be the reason for observing higher Z-average size in DLS measurements.

**Table 1:** Z-average (d.nm) PdI and zeta potential (mV) with standard deviation (SD). *ND*, not determined

Sample	Z-average size (SD) (nm)	Polydispersity index (SD)	Zeta potential (SD) (mV)
Blank	136.2 (2.06)	0.07 (0.01)	− 1.20 (0.12)
Pluronic F-68	158.4 (1.58)	0.13 (0.02)	ND
Cyclosporine A	162.3 (2.17)	0.06 (0.02)	4.84 (0.41)
Prednisolone	170.6 (0.37)	0.07 (0.02)	− 2.02 (1.01)
Curcumin	268.0 (4.24)	0.07 (0.05)	− 11.3 (0.66)

The zeta potential distribution of blank and drug-loaded nanoemulsions was measured to predict the stability and to elucidate the effect after drug loading (Table 1 and Fig. S-1). As shown in Table 1, the zeta potential was slightly shifted towards positive values after cyclosporine loading, whereas a significant shift in zeta potential towards negative values was observed after curcumin loading. However, no significant change was observed after prednisolone loading. These shifts can be attributed to the structure of the drugs where the presence of amine group (cyclosporine) and hydroxyl groups (curcumin) influence the overall surface charge. Generally, zeta potential values that exceed 30 mV (positive or negative) is ideal for an electrostatically stabilized colloidal system. However, the zeta values near to zero in this study indicates that that the nanoemulsions are stabilized sterically rather than electrostatically due to the presence of Pluronic PEO blocks at the surface of oil droplets. Therefore, the globule fusion in curcumin samples could be attributed to the slightly negative surface charge, which is neither high enough to produce a stable emulsion nor neutral due to presence of sufficient PEO blocks, thus being susceptible to aggregation.

### Drug encapsulation in nanoemulsions

**Fig. 6:**
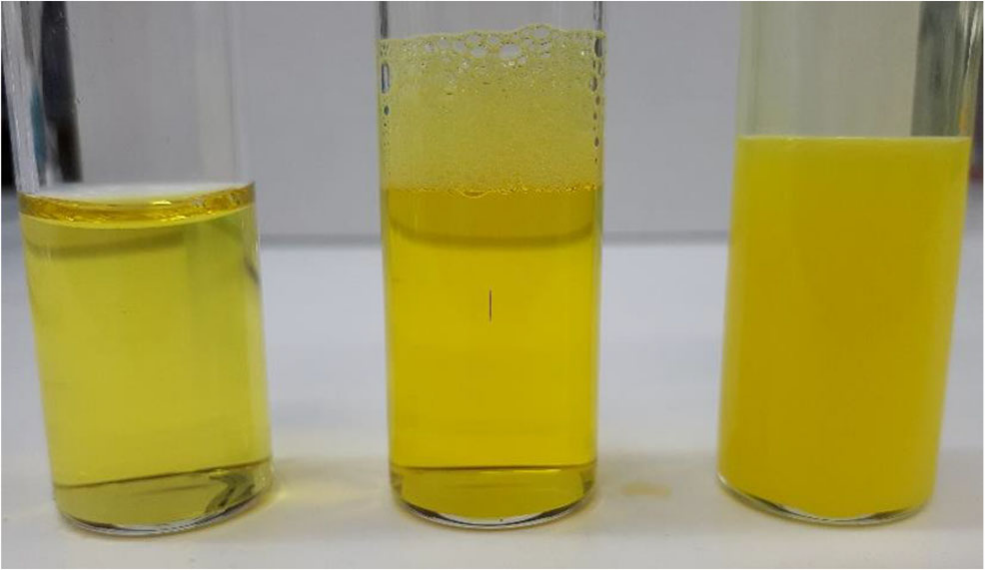
From left to right; curcumin in water, curcumin in Pluronic micelles and in nanoemulsion

**Fig. 7:**
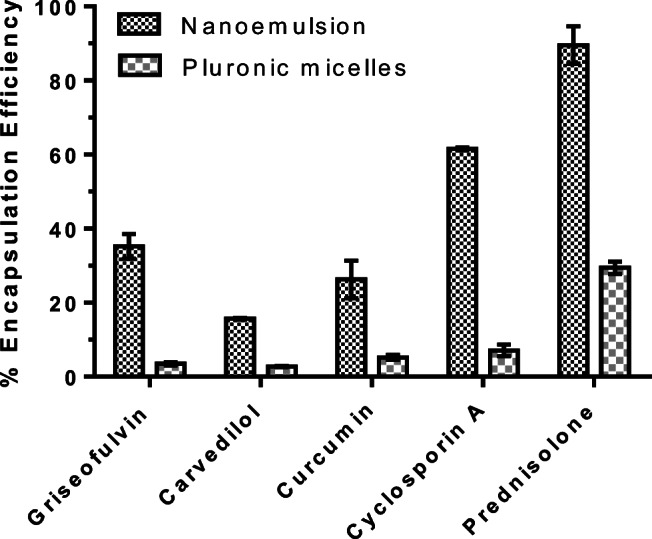
Percentage encapsulation efficiency of drugs in nanoemulsion and Pluronic micelles. Data represents average of three measurements with standard deviation

Nanoemulsions are widely investigated to enhance the aqueous solubility of drugs, and therefore, in this study, five different drugs with poor aqueous solubility have been explored to establish the usefulness of PDL nanoemulsion in enhancing drug solubility. It was expected that during nanoprecipitation, the hydrophobic drug is encapsulated within PDL droplets due to hydrophobic interaction. Therefore, the solubility of drug in PDL polymer could be a determining factor in solubility enhancement. To investigate this, all samples were filtered through 0.22-μ syringe filter in order to remove any precipitated drug, and the amount of drug was calculated spectrophotometrically or by HPLC. A typical appearance of sample after filtration is shown in Fig. 6. For comparison purposes, and to make sure that the increment in solubility is due to the presence of PDL polymer, the amount of drugs in Pluronic micelles samples were also calculated. As expected, PDL nanoemulsion was capable to enhance the aqueous solubility of all tested drugs and demonstrate superior performance compared with Pluronic F-68 micelles. The percentage drug encapsulation efficiency of drugs in both formulations is shown in Fig. 7. The results suggested that the nanoemulsion was capable to increase the aqueous solubility of drugs by three to ten times compared to Pluronic micelles.

No significant difference (*P* < 0.05, unpaired *t* test) in EE% was observed using Pluronic micelles between carvedilol-griseofulvin and curcumin-cyclosporine A. However, the EE% observed for nanoemulsion clearly demonstrates statistical difference among these comparisons. This observation undoubtedly suggests that PDL is playing a crucial role in enhancing aqueous solubility of drugs while Pluronic micelles have negligible effect in nanoemulsion formulations. From the results, it can be concluded that prednisolone demonstrate higher compatibility with PDL nanoemulsion while carvedilol showed least. However, it should be noted that the drug content is calculated in liquid dosage form, and thus the results are the combination of intrinsic solubility of drugs in water and drug-loaded in PDL nanoemulsion.

### Stability

**Fig. 8:**
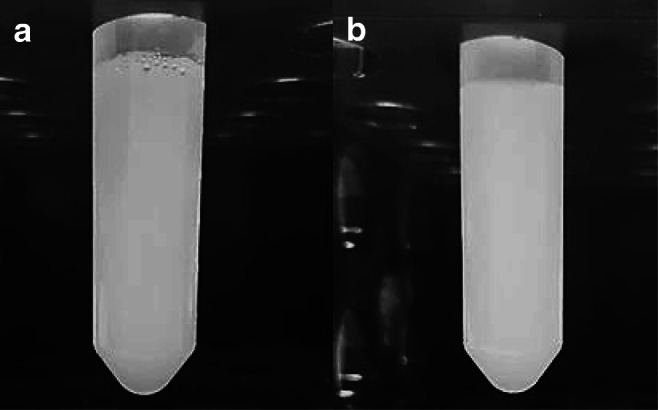
Nanoemulsion before **a** and after **b** centrifugation

Nanoemulsions are thermodynamically unstable, and thus they are likely to display creaming/separation on long-term storage. Thus, the stability of newly prepared polymeric nanoemulsion was determined by applying stress condition (centrifugation) to accelerate emulsion breakage. No sign of phase separation/creaming/sedimentation was observed after centrifuging the sample for 30 min at 10,000 rpm (Fig. 8). In addition, long-term storage stability was checked by keeping two drug-loaded nanoemulsion samples (prednisolone and cyclosporine A) for 3 months at room temperature and at 50 °C. Samples were withdrawn after every 30 days and analysed to observe the change in size and drug content.

**Fig. 9:**
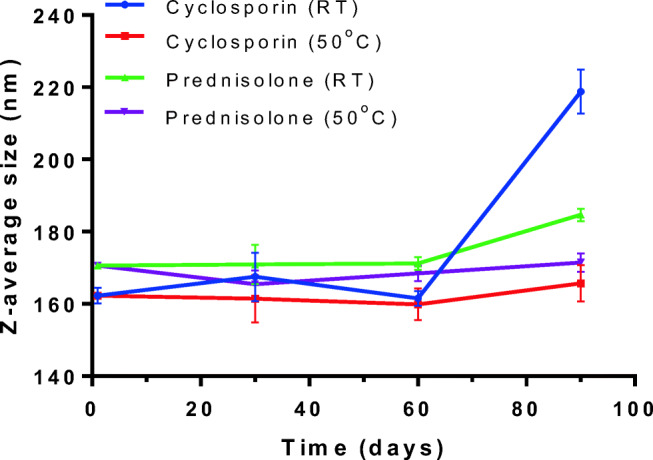
Change in size over 3 months for cyclosporine A and prednisolone stored at room temperature (RT) and at 50 °C. Data represents average of three measurements with standard deviation

No sign of phase separation or gravitational separation was observed for any of the samples upon visual inspection during 3 months of storage. However, as per DLS results, a significant change in size was observed after 2 months for samples stored at room temperature in contrast to samples stored at 50 °C samples (fig. 9). Generally, a change in size during accelerated stability studies is expected from sample stored at higher temperature. A previous report on stability of lemon oil nanoemulsions also suggested an increase in size at elevated temperatures (40 °C) due to coalescence of oil globules [33]. Size uniformity at higher temperature with polymeric nanoemulsion could be attributed to the melting point of Pluronic F-68 (50–55 °C), which probably makes the polymer more flexible at higher temperature. Although, the maximum size recorded is less than 220 nm with PDI less than 0.15. Thus, the results clearly suggest that the polymeric nanoemulsion are stable for at least up to 3 months in terms of size change, as no major change in size was observed (coalescence, aggregation).

**Fig. 10:**
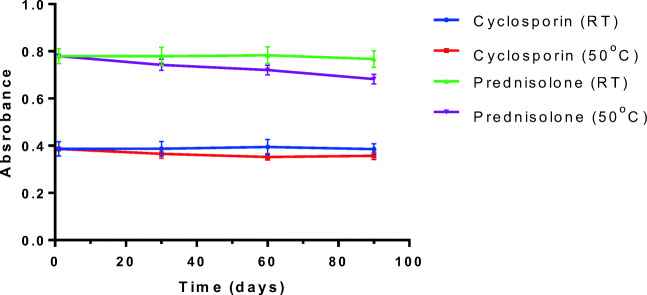
Stability from UV-Vis spectra for prednisolone and cyclosporine A stored at 50 °C and at room temperature (RT). Data represents average of three measurements with standard deviation

Additionally, to investigate the ability of nanoemulsion towards prevention of degradation of loaded drugs, drug concentration was monitored for 3 months. As expected, prednisolone concentration starts dropping just after 1 month of storage at 50 °C due to the higher intrinsic solubility of this drug in water, and thus a significant amount of unloaded drug is available in aqueous phase (Fig. 10). In contrast, no significant difference was observed for cyclosporine samples due to least availability of drug in water phase. This result clearly indicated that nanoemulsion is capable in protecting the loaded drug from degradation.

### Haemolysis

**Fig. 11:**
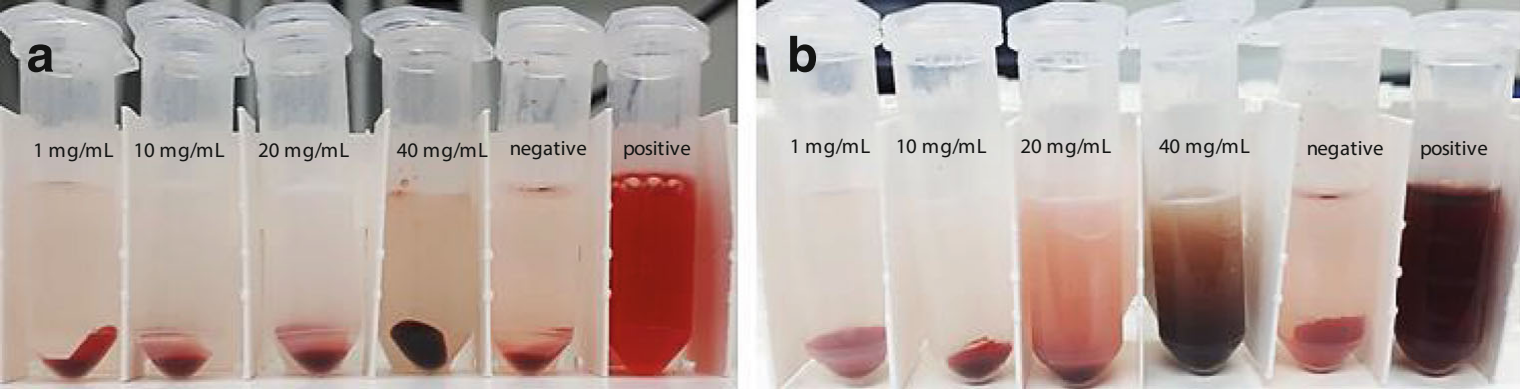
Appearance of centrifuged samples after incubation for **a** 1 h and **b** 24 h

**Fig. 12:**
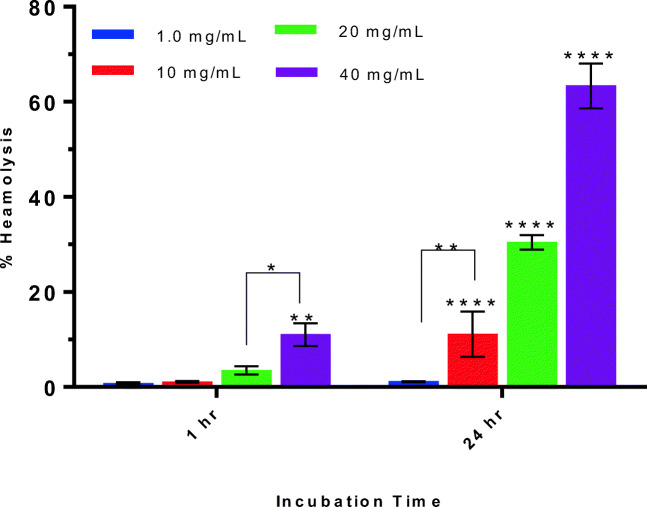
Percent haemolysis of RBCs observed with different concentration of nanoemulsion incubated for 1 h and 24 h at 37 °C. Data represents average of three measurements with standard deviation, and asterisk represents the significant difference level among the groups

Haemolysis is usually dose-dependent, as increasing concentrations of the test materials correspond with higher levels of haemolysis. Correspondingly, in this study, the percentage of haemolysis increased with the increasing concentrations of nanoemulsion. Approximately, 10% of heamolysis was observed after 1 hour of incubation of 40 mg/mL nanoemulsion with red blood cells (RBCs) whereas lower-tested concentration did not elicit haemolysis more than 5%. In contrast, after 24 h incubation, concentrations starting from 10 mg/mL is capable to rupture the RBCs above 10% (Figs. 11 and 12). The 2-way ANOVA, Sidak’s multiple comparison test was performed to determine the significant difference in groups between 1- and 24-h haemolysis results, and no significant difference was observed for 1.0 mg/mL sample. Rest of the samples were found significantly different to each other. Thus, from the results, it can be concluded that the nanoemulsion containing 1 mg/mL of PDL polymer is non-heamolytic and promising to be administered through IV route without eliciting any side effects [26].

### Cytocompatibility

**Fig. 13:**
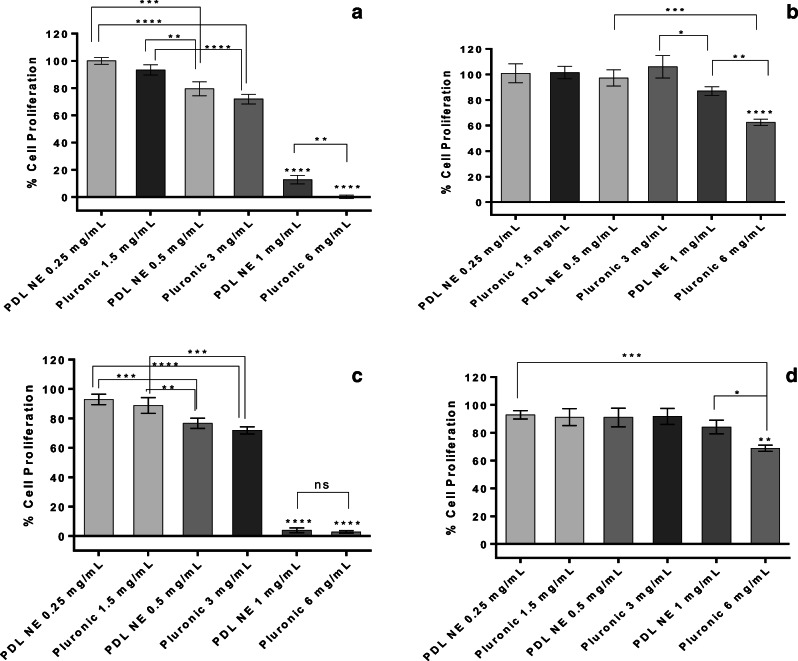
Percentage cell proliferation calculated by WST-1 assay after treatment with different samples on MDA-MB-231 cells at time point **a** 48 h and **c** 72 h and on MEF cells at time point **b** 48 h and **d** 72 h. Data represents average of three measurements with standard deviation and asterisk represents the significance difference level among the groups

The preliminary investigation of cytotoxicity of nanoemulsion and surfactant Pluronic F-68 was performed via cell viability assay on cancerous MDA-MB-231 and non-cancerous MEF cells. The percentage cell proliferation after treatment with different concentrations of nanoemulsion and Pluronic F-68 is shown in Fig. 13. It can be concluded from the results that the toxicity profile of PDL nanoemulsion is identical to the Pluronic samples. Both samples demonstrate concentration and time-dependent toxicity, and no significant difference was observed between nanoemulsion and Pluronic F-68 micelles except for 6 mg/mL concentration of Pluronic samples, which suggest that Pluronic micelles could be the main cause of toxicity at higher concentrations. To better understand the toxicity profile of PDL nanoemulsion and the role of Pluronic F-68, samples with the ratio of polymer to Pluronic (i.e. 1:2) was tested (Fig. S-2).

Even after reducing the Pluronic concentration, we observed the same trend of toxicity, and thus it can be concluded that PDL does not induce any cytotoxicity by itself in the tested formulations, and the presence of Pluronic F-68 is the probable reason for cell death at higher concentrations. However, these results clearly suggest that the cytotoxicity of Pluronic F-68 surfactant at higher concentration (6 mg/mL) is reduced to a great extent when PDL polymer was loaded in the core (Fig. 13) except for the 72 h sample in MDA-MB-231 cells. This could be related to the decrease in critical micelle concentration (CMC) of Pluronic F-68 in the presence of hydrophobic polymer, which induced hydrophobic interaction [33–35]. The toxicity of surfactants is usually associated with the amount of surfactant monomer present below CMC, and thus decrease in CMC can be directly related to decrease in toxicity [36]. Furthermore, we have observed that Pluronic F-68 is more toxic to the cancer cells compared with the tested normal cell line.

**Fig. 14:**
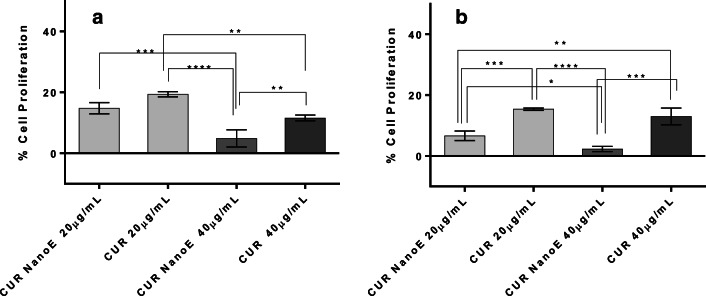
Percentage cell proliferation (MDA-MB-231) calculated by WST-1 assay after treatment with different curcumin samples at time point **a** 48 h and **b** 72 h. Data represents average of three measurements with standard deviation and asterisk represents the significant difference level among the groups. (CUR NanoE- curcumin-loaded nanoemulsion, CUR- pristine curcumin)

In addition, we evaluated the anticancer property of curcumin on MDA-MB-231 cancer cells in nanoemulsion form. The pristine curcumin cytotoxicity found on MDA-MB-231 cells is similar to the previously reported results [37]. The toxicity results suggests that the curcumin nanoemulsion formulation demonstrate a slightly higher toxicity, notably after 72 h of incubation compared to free curcumin (Fig. 14). The superior toxicity of curcumin in nanoemulsion can be attributed to the enhanced stability of loaded curcumin compared with free drug, which in turn exhibits higher toxicity [26].

## Conclusion

In this study, nanoemulsion was successfully fabricated using hydrophobic oily polymer PDL instead of oil, and Pluronic F-68 as surfactant. The droplet size found was less than 200 nm for all drug-encapsulated nanoemulsion samples, except for the curcumin sample where possible coalescence is expected based on the acquired surface charge. Drug encapsulation results suggests that the nanoemulsion samples were able to increase the aqueous solubility of all tested drugs by 3–10 times compared with Pluronic micelles. Polymeric nanoemulsions showed good stability under stress condition (centrifugation) and upon long-term storage at room temperature and 50 °C in terms of size, coalescence/creaming and drug degradation. The haemolysis and cytotoxicity studies suggest that the nanoemulsions are non-haemolytic up to concentrations of 1 mg/mL, and PDL polymer does not contribute in augmenting cell death. This preliminary study clearly provided an indication that stable nanoemulsions with adequate drug loading can be prepared via a facile method using PDL polymer. However, further studies are warranted for the improvement of polymeric nanoemulsions in terms of clinical translation.

## Electronic supplementary material

ESM 1 (DOCX 55 kb)
