# Supplementary material for: Facile methodology of nanoemulsion preparation using oily polymer for the delivery of poorly soluble drugs
Source: Drug Deliv Transl Res. 2019 Dec 19;10(5):1228–40. doi: 10.1007/s13346-019-00703-5 (PMC7447668; doi:10.1007/s13346-019-00703-5)
Supplement: Supplementary file 1 — (DOCX 55 kb) [file 13346_2019_703_MOESM1_ESM.docx]

**Supporting Information**

**Facile methodology of nanoemulsion preparation using oily polymer for delivery of poorly soluble drugs**

Johanna Wik ^1^, Kuldeep. K. Bansal ^1, 2^*, Tatu Assmuth ^2^, Ari. Rosling ^2^, Jessica. M. Rosenholm ^1,^*

^1^ Pharmaceutical Sciences Laboratory, Faculty of Science and Engineering, Åbo Akademi University, 20520 Turku, Finland

^2^ Laboratory of Polymer Technology, Centre of Excellence in Functional Materials at Biological Interfaces, Åbo Akademi

University, Biskopsgatan 8, 20500 Turku, Finland

Figure S-1 Zeta potential (mV) distribution of various nanoemulsion (NE) formulations (A) Blank NE, (B) Prednisolone loaded NE (C) cyclosporin A loaded NE and (D) curcumin loaded NE.

Figure S-2 Percentage cell proliferation (MDAMB-231) calculated by WST-1 assay after treatment with different samples at time point (A) 48 h and (B) 72 h. Data represents average of three measurements with standard deviation and asterisk represents the significance difference level among the groups. Each tested concentration of nanoemulsion corresponds to Pluronic F-68 are not significant to each other.
